# Correction: Lymphatic transport in anti-tumor immunity and metastasis

**DOI:** 10.1084/jem.2023195403262025c

**Published:** 2025-04-04

**Authors:** Mengzhu Sun, Julien Angelillo, Stéphanie Hugues

Vol. 222, No. 3 | https://doi.org/10.1084/jem.20231954 | February 19, 2025

The authors regret that, in their original article, the reference Johnson et al. (2017) was incorrectly cited. In addition, Fig. 2 b and its legend have been corrected to include the axis LYVE-1:hyaluronan, which shows receptors mediating DC entry to LVs.

The corrected paragraphs are shown here, with the added text in bold. Both the original and corrected Fig. 2 are shown here, as is the updated figure legend with the added sentence in bold.

The errors appear in print and in PDFs downloaded before March 27, 2025.

## LECs as immune regulators

### Lymphatic transport of immune cells (second paragraph)

Under CCL21 guidance, CCR7+ DCs migrate from the tissues to the lymphatic capillaries (Russo et al., 2016; Tal et al., 2011; Weber et al., 2013). Interestingly, steady-state DCs autonomously migrate into the LVs mainly by flowing and squeezing (Lämmermann et al., 2008) (Fig. 2 a). The sole force of actin-network expansion promotes protrusive flowing of the leading edge, whereas the myosin II–dependent squeezing contraction of the trailing edge propels the rigid nucleus and allows the passage through narrow gaps, which is sufficient to drive DC locomotion (Lämmermann et al., 2008). This process only becomes integrin** receptor/ligand** dependent under inflammatory conditions when LECs upregulate ICAM-1 and VCAM-1**, such as through integrin and ICAM-1/VCAM-1 interactions** (Arasa et al., 2021; Johnson et al., 2006) (Fig. 2 b)**, or via LYVE-1/hyaluronan (HA) axis–mediated transmigration (Fig. 2 b) (Johnson et al., 2017).** Recent findings show that in case of inflammation, the connective tissue membrane surrounding the lymphatic collectors is subjected to degradation, whereas the collectors upregulate the DC-trafficking molecule VCAM-1, facilitating DC penetration and rapid transportation to draining LNs (Arasa et al., 2021) (Fig. 2 c). After reaching the LNs, DCs first enter the LN subcapsular sinus. Ceiling LECs produce ACKR4 (or CCRL1), a decoy receptor for CCL21 and CCL19, that scavenges these chemokines in the sinus lumen and shapes chemokine gradients directed toward the floor layer. Some studies indicate that in some contexts, CCR7 may not be sufficient for proper DC positioning within the LN and that other chemokine networks may be involved in subset-specific DC migration to LNs. For example, Th2-skewing stimulus efficiently induces the migration of CD301b^+^ DCs into draining LNs despite low surface CCR7 levels. Instead, CD301b^+^ DCs upregulate CCR8, and their migration toward the LN parenchyma is facilitated by floor LECs that produce CCL1 (Qu et al., 2004; Sokol et al., 2018) (Fig. 2 d).

### Direct regulation of immune cells by LECs

Apart from supporting cell transportation, LVs actively contribute to immunosurveillance and immunomodulation. Human and mouse LN LECs express MHC class I (MHC-I) and MHC-II. At steady state, LN LECs endogenously express peripheral tissue–restricted antigens that they can present through MHC-I (**Johnson et al., 2017;** Olszewski, 2005), inducing the deletion of autoreactive CD8^+^ T cells and therefore participating in peripheral T cell tolerance (Cohen et al., 2010; Fletcher et al., 2010; Tewalt et al., 2012). LECs can also cross-present exogenous antigens via MHC-I to induce antigen-specific CD8^+^ T cell apoptosis (Hirosue et al., 2014). Although LECs seem unable to present antigens through MHC-II due to the absence of the H2-M expression at steady state (Rouhani et al., 2015), they can induce antigen-specific CD4^+^ T cell tolerance by presenting peptide–MHC-II complexes acquired from DCs or by presenting peptides through IFN-γ–induced endogenous MHC-II under inflammatory condition (Dubrot et al., 2014). For example, MHC-II deficiency in LN LECs promotes defective Tregs and increased effector CD4^+^ T cells, resulting in impaired peripheral CD4^+^ T cell tolerance and enhanced autoantibody production in aging mice (Dubrot et al., 2018). Furthermore, S1P produced by LN LECs supports naive T cell survival through the stimulation of their mitochondrial function (Mendoza et al., 2017). LECs express IL-7 in both LN and peripheral tissues, suggesting that they might support T cell survival (Hara et al., 2012). Under infection and inflammation, some factors released into the surrounding tissue activate LECs to induce their proliferation, a process called lymphangiogenesis. These factors, named lymphangiogenic factors, promote LEC proliferation, sprouting, and activation, altogether increasing the abundance of LVs in inflamed tissues. LVs experience remarkable expansion, accompanied by a transient increase of lymph flow, promoting the translocation of DCs and lymphocytes to the LN parenchyma and therefore the initiation of the immune response. Extranodal lymphangiogenesis is accompanied by intranodal lymphangiogenesis in the LNs draining the inflamed tissue, which can be reverted by a T cell–dependent IFN-γ–mediated negative regulation (Kataru et al., 2011). Upon inflammatory stimuli, LN LECs further express immunosuppressive enzymes, such as indoleamine dioxygenase and inducible nitric oxide synthase (iNOS), thereby suppressing T cell proliferation and activation (Lukacs-Kornek et al., 2011), as well as inhibiting DC maturation (Christiansen et al., 2016). During viral infection and vaccination, LN LECs function as antigen-archiving cells and slowly release these antigens into the LN parenchyma to contribute to protective immune responses (Kedl et al., 2017; Tamburini et al., 2014). During bacterial infection, such as *Mycobacterium tuberculosis*, the bacteria can be transported and internalized by LN LECs, in which bacterial proliferation is inhibited by the production of nitric oxide by LECs when exposed to IFN-γ (Lerner et al., 2016). Tumors are a typical condition where lymphangiogenesis occurs andwhere some of the remodeling of LEC features described above might apply, together with specific adaptations induced by the tumor microenvironment (TME) (developed in Lymphangiogenic factors in the TME).

**Figure:**
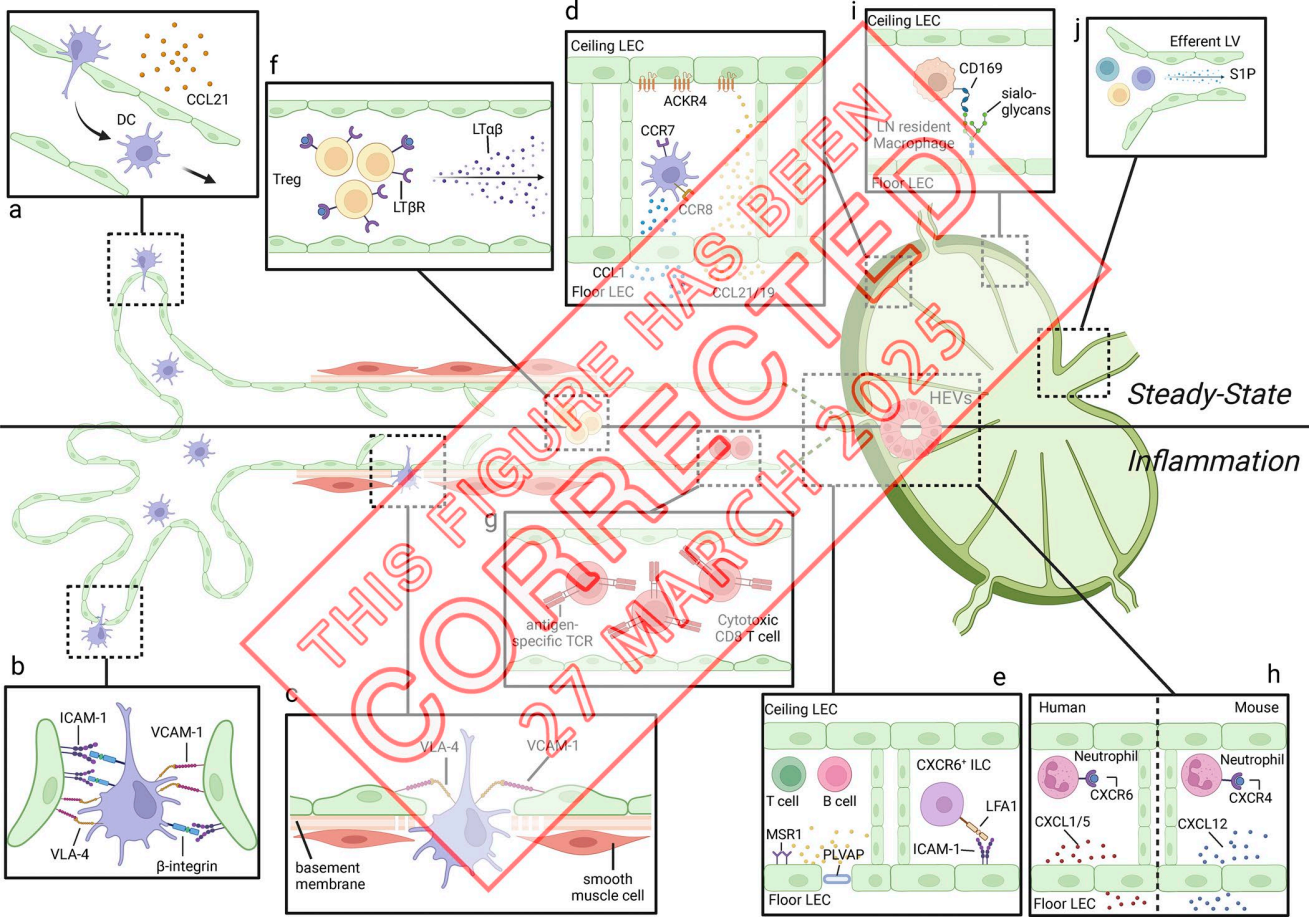


**Figure 2.**
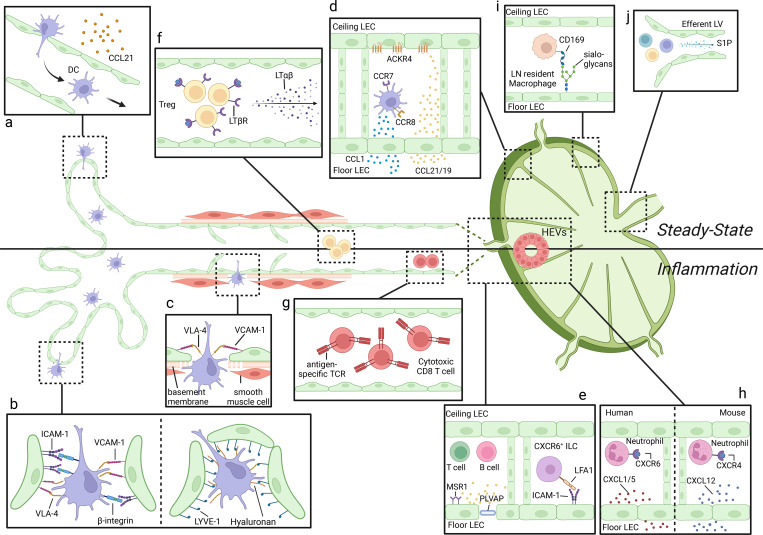
Immune cell transportation through LVs. (a) At steady state, DCs migrate and squeeze into LVs under CCL21 guidance. (b) This process is facilitated by the interaction of ICAM-1/β-integrin and VCAM-1/VLA-4 molecules, respectively, expressed at LEC and DC surface under inflammation conditions. **Migrating DCs can also assemble endogenous hyaluronan surface coat in order to dock to the lymphatic endothelium via lymphatic specific marker LYVE-1 during their transmigration to the lymphatic lumen.** (c) Upon inflammation, DCs can also take “shortcuts” to enter directly into lymphatic collectors via VCAM-1/VLA-4 interactions, allowing a faster migration to LNs. (d) In the LNs, ceiling LECs produce the scavenger chemokine ACKR4 in the sinus lumen and shape gradients toward the LN floor layer, while floor LECs secrete CCL1 to further facilitate CCR8^+^ DC migration toward the LN parenchyma. (e) Although naive lymphocytes mostly enter LNs through HEVs, activated T cells and memory B cells penetrate the LNs through LVs and reach the T cell zone following CCL21 gradients, while LN-resident CXCR6^+^ innate-like lymphoid cells position through CCL20 and migrate across the sinus via LFA-1/ICAM-1 interactions. (f) Treg cells represent the most abundant cell type found in the afferent LVs. (g) In tumor context, antigen-experienced CD8^+^ T cells can be directly transferred from peripheral tissues to draining LNs via afferent LVs. (h) In humans, CXCL1 and CXCL5 expressed by LECs in the subcapsular sinus floor and medullary sinuses seem related to the migration of neutrophils in the LNs. The mechanism is different inmice, with neutrophils entering inflamed LNs through the CXCR4/CXCL12 axis. (i) Floor LECs regulate the localization of LN-resident CD169^+^ macrophages through the expression of sialoglycans. (j) LECs near cortical sinuses, lining efferent lymphatics, or lining LV capillaries in peripheral tissues S1P, allowing the exit from LNs of S1PR1^+^ immune cells. LFA-1, LFA molecule-1; PLVAP, plasmalemma vesicle–associated protein; ILC, innatelike lymphoid cells.

